# Differentiated osteoblasts derived decellularized extracellular matrix to promote osteogenic differentiation

**DOI:** 10.1186/s40824-018-0115-0

**Published:** 2018-02-23

**Authors:** Jin Jeon, Min Suk Lee, Hee Seok Yang

**Affiliations:** 1 Department of Nanobiomedical Science & BK21 PLUS NBM Global Research Center for Regenerative Medicine, Dankook University, Cheonan, 330-714 Republic of Korea; 2 Department of Pharmaceutical Engineering, Dankook University, Cheonan, 330-714 Republic of Korea

**Keywords:** Cell-derived extracellular matrix, Decellularization, Osteogenic differentiation

## Abstract

**Background:**

The extracellular matrix (ECM) can directly or indirectly influence on regulation of cell functions such as cell adhesion, migration, proliferation and differentiation. The cell derived ECM (CD-ECM) is a useful in vitro model for studying the comprehensive functions of CD-ECM because it maintains a native-like structure and composition. In this study, the CD-ECM is obtained and a test is carried out to determine the effectiveness of several combinations of decellularized methods. These methods were used to regulate the optimal ECM compositions to be induced by osteogenic differentiation using primary isolated osteoblasts.

**Result:**

We investigated the effect of osteoblasts re-seeded onto normal osteoblast ECM under the growth medium (GM-ECM) and the osteogenic differentiation medium (OD-ECM). The osteoblasts were then cultured statically for 1, 2, and 4 weeks in a growth medium or differentiation medium. Before osteoblast culture, we performed immunostaining with filamentous actin and nuclei, and then performed DNA quantification. After each culture period, the osteogenic differentiation of the osteoblasts re-seeded on the OD-ECMs was enhanced osteogenic differentiation which confirmed by alkaline phosphatase staining and quantification, Alizarin Red S staining and quantification, and von Kossa staining. The OD-ECM-4 W group showed more effective osteogenic differentiation than GM-ECM and OD-ECM-2 W.

**Conclusions:**

The OD-ECM-4 W has a better capacity in a microenvironment that supports osteogenic differentiation on the GM-ECM and OD-ECM-2 W. The ECM substrate has a wide range of applications as cell culture system or direct differentiation of stem cell and excellent potential as cell-based tissue repair in orthopedic tissue engineering.

## Background

Extracellular matrix (ECM) is specialized architecture composed of extracellular proteins which are known to interact with various cells and influence the regulation of cell behaviors such as cell adhesion, migration, proliferation and differentiation [1–3]. ECM is composed of diverse molecules such as collagen, fibronectin and other proteins that are interlaced with proteoglycans [4]. The composition and structure of ECM can be changed by the phenotype of the resident cells and the function of the tissues or organs. In turn, the ECM can affect the phenotype and behavior of the resident cells [5–7]. Moreover, the ECM can modulate the signal transduction activated by various bioactive molecules, such as growth factors and cytokines [8].

The ECM from tissues or whole organs has been studied as biomaterials which comprises intestinal submucosa, heart valve, blood vessel, skin, nerve, tendon, ligament, urinary bladder, vocal fold, amniotic membrane, heart, liver, and lung in tissue engineering and regenerative medicine [9–16]. Tissue-derived decellularized ECM (TD-ECM) obtained from tissues have properties that preserve the structures of their respective tissues. However, they may have several problems such as tissue scarcity, host responses, and pathogen transfer [17–19]. Recently to address these problems, many studies have been carried out using ECM derived from cultured cells. Cell-derived ECM (CD-ECM) from cultured cells has several advantages over TD- ECM. In the CD-ECM, it is easy to eliminate pathogen transfer and maintain pathogen-free condition. The CD-ECM also provides the desired geometry and porosity without the limitation of poor cell penetration. Moreover, the CD-ECM can be derived from autologous cells to make autologous CD-ECM scaffolds [20, 21].

The CD-ECM contains specific molecules secreted by the cells as well as growth serum proteins during proliferation. The composition of CD-ECM molecules can change according to the differentiation medium composition. Thus, our approach involves the development of more osteoinductive culture conditions that impact the ability of differentiated CD-ECMs to induce re-seeded cell functions. Osteogenic differentiated ECM (OD-ECM) is used to produce collagen type-I, fibronectin, biglycan and decorin. The collagen type-I can not only upregulated alkaline phosphatase (ALP) and osteopontin (OPN), but the decorin and biglycan also influence differentiation of osteoblasts [22–26]. In addition to the preparation of OD-ECM, hydroxyapatite (HA) has been deposited during osteoblasts maturation. The development of OD-ECM involved osteoconductive HA of native components which induced cellular differentiation. The ALP activity and messenger RNA levels of osteoblasts cultured in HA surface were increased at the early stage of osteogenic differentiation, and osteocalcin expression was also increased at the late stage [27, 28].

The aim of this study was to investigate the effect of different compositions of OD-ECM by different stages of osteogenesis. We cultured confluent osteoblasts on a tissue culture plate. The osteoblasts were then decellularized after treatment of differentiate medium to prepare for the different stages of OD-ECM. They were treated during 2 and 4 weeks. We investigated the re-seeded osteoblast effect on different compositions of ECM as followed: GM-ECM (normal osteoblast ECM), OD-ECM-2 W (osteogenic differentiated ECM during 2 weeks culture), and OD-ECM-4 W (osteogenic differentiated ECM during 4 weeks culture). Differentiation and maturation of re-seeded osteoblasts were determined by analysis of the known indicators of the osteoblast phenotype, calcification, mineralization, and protein activity under a growth medium and osteogenic differentiation medium.

## Methods

### Osteogenic differentiation with primary isolated osteoblast

The primary isolated rat osteoblasts were obtained from neonatal rat (1–2 day old, IACUC approved number: DKU-16-026). The calvarias of neonatal rat were carefully dissected to extract neonates, and washed to use Hank’s balanced salt solution with 1% penicillin/streptomycin (PS, Corning, NY, USA). The washed calvaria was chopped and immersed in the digest solution (0.25% trypsin, collagenase type-II of 1 mg/mL), and then treated at 5, 15, and 25 mins under incubation at 37 °C. The digest solution treated at 5 mins after the supernatant of the first digest solution was discarded. After 15 and 25 mins, the supernatant of the digest solution was accumulated to centrifuge at 5 mins at 1500 rpm. The supernatant of the digest solution was suctioned and re-suspended using Dulbecco’s modified Eagles media (DMEM, Corning, NY, USA) with 10% fetal bovine serum (FBS, Corning, NY, USA) and 1% PS. The resuspended solutions were filtered using 70 μm nylon filters (BD Biosciences). The primary isolated rat osteoblast was cultured using DMEM with 10% FBS and 1% PS at 37 °C and 5% CO_2_ conditions. Osteoblasts (Passage number 4) were cultured on tissue culture polystyrene (TCPS) plates for 3 days with growth medium (GM) or osteogenic differentiation medium (ODM) which consisted of 100 nM of dexamethasone (Sigma-Aldrich), 50 μM of L-ascorbic acid (Sigma-Aldrich), 10 mM of β-glycerophosphate (Sigma-Aldrich) and 7 mM of L-glutaminein (Sigma-Aldrich) for 2 and 4 weeks to prepare OD-ECM.

### Preparation of various decellularized ECM substrates

Osteoblasts were cultured on TCPS plates in growth medium with or without osteogenic induction factors. The prepared decellularization solution (D-solution) was comprised of KCl (0.5 M, 1.5 M, and 2.0 M) and Triton X-100 (TX, 0.05%, 0.1%, and 0.2%) in a 50 mM of tris-buffer (pH 8.0). D-solution was sterilized using a syringe filter (0.45 μm, Corning, NY, USA). After each time points, the cultured layer was gently washed twice with phosphate buffered saline (PBS), was then immersed in sterilized D-solutions to remove cellular components, and then shaken gently for 1 h. The decellularized matrix was washed 6 times very carefully with 10 mM tris-buffer (pH 8.0) and subsequently rinsed 3 times with PBS. The decellularized matrix was observed using an optical microscope (IX71, Olympus, Tokyo, Japan). We divided three types of decellularized ECM matrices using different methods: (i) the osteoblast was cultured with growth medium for 3 days as GM-ECM.; (ii) the osteoblast cultured with osteogenic differentiation medium for 2 and 4 weeks as OD-ECM-2 W and (iii) OD-ECM-4 W.

### Confirmation of various decellularized ECM

Cultured cells and decellularized ECM were fixed with 4% paraformaldehyde solution for 15 mins at room temperature. The fixed cells and decellularized ECM were gently washed 3 times with PBS. The washed cells and decellularized ECM were immersed in 0.2% Triton X-100 in PBS for 10 mins. The immersed cells and decellularized ECM were treated with 5% bovine serum albumin solution (Sigma-Aldrich) to block the non-specific binding of antibodies at room temperature for 1 h. The blocked cells and decellularized ECM were then incubated with primary mouse specific antibodies, which were diluted for actin fibers (1:40, Alexa 488-conjugated phalloidin, Invitrogen) at room temperature for 20 mins and washed gently 3 times with PBS. Then, the phalloidin stained cells and decellularized ECM were counter-stained with DAPI (4′,6-diamidino-2-phenylindole, Vector Laboratories). To visualize laminin on ECM, blocked cells and decellularized ECM were incubated with primary antibodies of 1:200 diluted mouse anti laminin (Abcam) at 4 °C for overnight. After reaction with the primary antibodies, the cells were washed gently 3 times with PBS, and then incubated with secondary antibodies of diluted Rhodamine B (1:200, Jackson Immuno Research Laboratories) for 1 h at room temperature under dark condition. After brief washing with PBS 3 times, the cells and decellularized ECM were counter-stained with DAPI. The stained cells were observed under a confocal microscope (Carl Zeiss LSM 700, Oberkochen, Germany). To calculate the ECM area and all statistics was using Origin Pro (Origin Lab).

To confirm the complete removal of DNA, we performed DNA contents analysis. Cells and decellularized ECM were washed gently 3 times with PBS. The washed cells and decellularized ECM were lysed with 1% NP-40 solution (Sigma-Aldrich) for 2 h at 4 °C. The lysates were collected as supernatant and diluted to 20 μL/96 well plate, to which was added Tris-EDTA (TE) buffer of 80 μL and 1:200-diluted picogreen (Sigma-Aldrich). The DNA was then quantified at an absorbance of 520 nm using a plate reader (Spark 20 M multimode microplate reader, TECAN, Mannedorf, Switzerland).

### Re-seeded and cultured osteoblast on various decellularized ECM substrate

To confirm the improvement of osteogenesis on the decellularized matrix, primary isolated osteoblasts were re-seeded and cultured on various decellularized ECM, on which decellularization was performed after progressing osteogenic differentiation for 2 and 4 weeks. The osteogenic differentiation capacities of re-seeded osteoblasts were determined at 1, 2, and 4 weeks by analyzing the ALP activity and mineralization. ALP is a marker generally used for early osteogenic differentiation. The re-seeded and cultured osteoblasts in each group were washed gently with PBS, fixed with 4% paraformaldehyde solution for 1 min at room temperature, and then incubated with BCIP/NBT substrate solution (Sigma-Aldrich) for 20 mins at room temperature under dark condition. The stained cells on each group were gently washed 3 times with PBS and observed using an image scanner (V37-V370, EPSON, Seoul, Korea). To quantify the ALP activity, the cultured cells in each group twice washed with PBS. The cells were lysed with 1% NP-40 solution for 2 h at 4 °C. The cell lysates were then collected as supernatant and incubate with pNPP substrate (Sigma-Aldrich) for 30 mins at 37 °C. Then, 0.5 N NaOH solution was added as stop solution and quantified at an absorbance 405 nm using the plate reader. To normalize ALP activity, the supernatant was diluted 20 μL/96 well and a TE buffer of 80 μL, 1:200-diluted picogreen (Sigma-Aldrich) was then added. The ALP activity was quantified at absorbance 520 nm to use plate reader.

To perform the Alizarin red S stain, cells on each group were fixed with 4% paraformaldehyde solution (Sigma-Aldrich) for 15 mins, and then gently washed 2 times with PBS. The fixed cells were then stained with 2% Alizarin red S (Sigma-Aldrich) for 15 mins and washed 8 times. The stained cells were observed under an optical microscope. To quantify Alizarin red S staining, the stained cells were lysed in 10% acetic acid (Sigma-Aldrich) at room temperature for 30 mins. The cell lysates were then collected as supernatant after centrifugation at 15,000 rpm for 15 mins, and were then quantified using plate reader at absorbance 405 nm.

To perform the von Kossa stain, cells in each group were fixed with 4% paraformaldehyde for 15 mins at room temperature. The fixed cells were gently washed 3 times with distilled water and then stained with 5% silver nitride staining (Sigma-Aldrich) under UV radiation for 30 mins at room temperature. The stained cells were then washed 2 times with distilled water and observed using an image scanner.

### Statistical analysis

Values are expressed as means ± standard deviations. Statistical analysis was performed using Student’s *t*-tests. Results with *p* values of less than 0.05 were considered significant.

## Results

### Optimization of decellularization of osteoblast to fabricate extracellular matrix

**Fig. 1:**
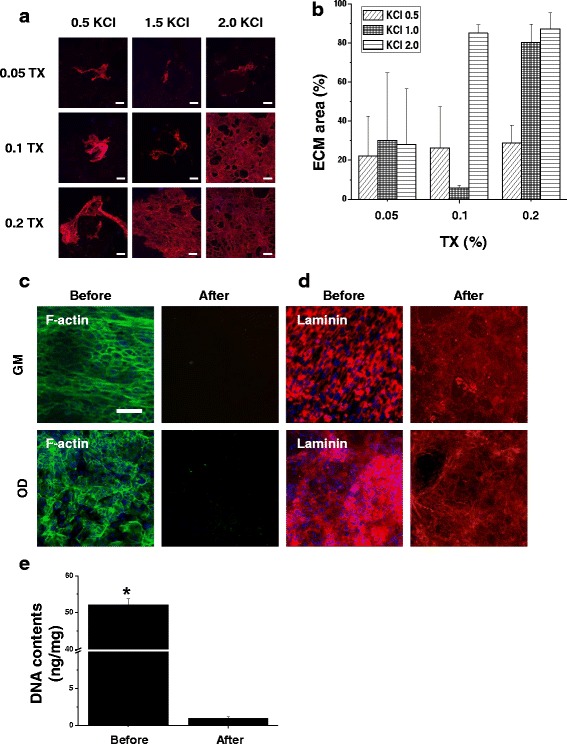
Confirmation and characterization of decellularized ECM. **a** Representative images of immunofluorescent staining with anti-laminin after decellularization with various concentrations of D-solutions. The scale bars represent 100 μm. **b** Quantification of remaining laminin after decellularization with various concentrations. **c** Representative images of immunofluorescent staining under GM and OD with anti-f-actin and (**d**) laminin before and after decellularization. The concentration of D-solution was KCl of 2.0 M and Triton X-100 of 0.2%. The scale bars represent 100 μm. **e** Quantification analysis of amount of residual DNA contents before and after decellularization. ^*^p < 0.05 compared to after decellularization group

The optimized decellularization was determined by diversifying concentration of the KCl and Triton X-100 solutions to remove the cellular components selectively from the matrices. To confirm the remaining ECM and removal of cellular components, laminin component of ECM and the nuclei were stained after decellularization. None of the groups showed staining of nuclei with DAPI (Fig. 1a). The concentration of decellularized solution and various methods, we confirmed the concentration of 2.0 M KCl and 0.2% Triton X-100 had the largest ECM composition and quantified positive laminin stained ECM after decellularization (Fig. 1a and b). The filamentous actin and nuclei were stained with 0.2 M KCl and Triton X-100 at concentrations of 2.0 and 0.2%, respectively, to clearly confirm the removal of the cellular components and nuclei. After decellularization, no filamentous actin and nuclei could be observed, indicating that the cellular components and nuclei had been completely removed (Fig. 1c and d). The DNA contents before decellularization were confirmed to be 52 ng/mg (Fig. 1e). However, the DNA contents significantly decreased to 0.9 ng/mg after the decellularized step. These data demonstrated the complete removal of cellular content and DNA after decellularization.

### Optical microscope images of various ECM and re-seeded osteoblasts

**Fig. 2:**
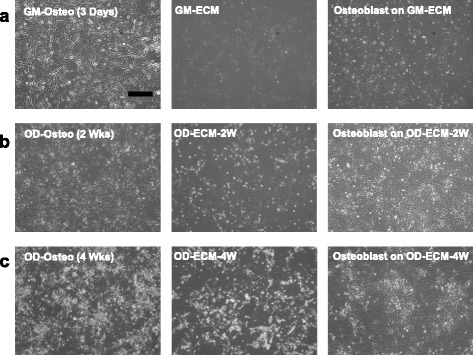
Representative optical microscope images of cultured osteoblast and re-seeded osteoblast on decellularized ECM with various conditions. **a** Osteoblasts were cultured with growth medium for 3 days (GM-Osteo) before decellularization. After decellularized ECM (GM-ECM). Osteoblasts were re-seeded on GM-ECM. Osteoblasts were cultured with osteogenic differentiation medium for (**b**) 2 weeks (OD-Osteo (2 Wks)) and (**c**) 4 weeks (OD-Osteo (4 Wks)). After decellularized ECM (OD-ECM-2 W and OD-ECM-4 W). Osteoblasts were re-seeded on decellularized OD-ECM. The scale bar represents 400 μm

Phase contrast optical microscopy showed morphology of osteoblast cultured on growth medium at 3 days (GM-Osteo) and osteogenic differentiation medium for 2 (OD-Osteo) and 4 weeks (OD-Osteo). To investigate the effect of ECM induced at different times, we prepared three ECM substrates: GM-ECM, OD-ECM-2 W, and OD-ECM-4 W. The different culture conditions showed that the three ECM substrates preserved their proliferation and differentiation potential. The decellurized ECM showed a network pattern and OD-ECM-2 W showed a slightly nodular mineralized pattern on the ECM network. The OD-ECM-4 W showed a diffuse mineralized pattern on the ECM network. After re-seeding osteoblast on three types of ECM matrices, the osteoblasts showed confluent proliferation and different morphologic characteristics were not observed among these groups (Fig. 2).

### Effect of re-seeded osteoblast on decellurized ECM matrix by ALP activity and quantification

**Fig. 3:**
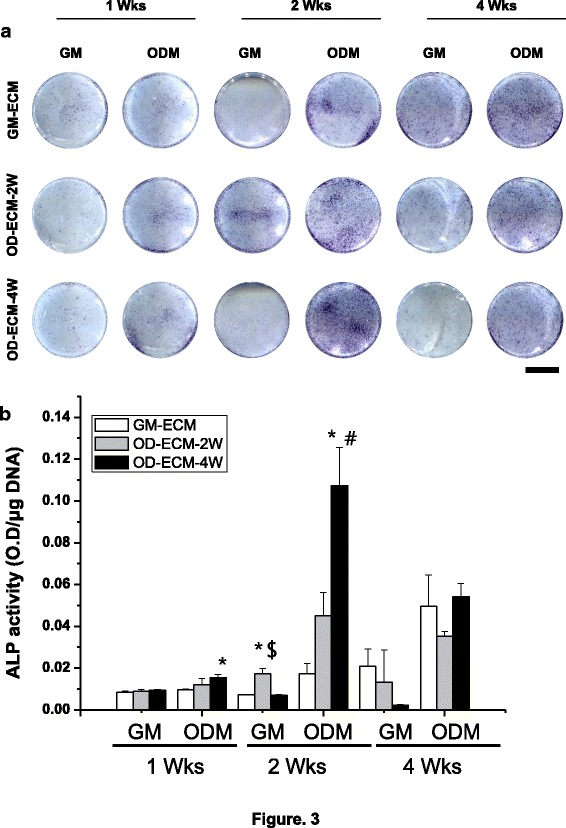
ALP staining and quantification on GM-ECM, OD-ECM-2 W, and OD-ECM-4 W for 1, 2 and 4 weeks with growth media or osteogenic differentiation media. **a** Representative images of ALP staining with each group. The scale bar represents 10 mm. **b** Quantification of ALP activity with each group. ^*^p < 0.05 compared to GM-ECM, ^*#*^*p* < 0.05 compared to OD-ECM-2 W, ^*$*^*p* < 0.05 compared to OD-ECM-4 W. GM, growth medium; ODM, osteogenic differentiation medium

The ALP activity of re-seeded osteoblasts was monitored over all periods of culture on GM-ECM, OD-ECM-2 W, and OD-ECM-4 W (Fig. 3a). The ALP activity, histochemical staining, and quantified ALP activity of the three different groups were examined for each time point. ALP is widely distinguished at the early stage of osteogenic differentiated biochemical marker for calcium phosphate. The ALP activity of all groups showed no significant difference at 1 week. However, the ALP activity of OD-ECM-4 W with the differentiation medium group was significantly more increased than that of other groups at 2 weeks. The all-around area of the cultured cells was stained with ALP on OD-ECM-4 W, and the result indicated that the re-seeded osteoblast activity was highly activated at 2 weeks. It was obvious that ALP staining on OD-ECM-4 W with differentiation medium was stronger than that on GM-ECM and OD-ECM-2 W with differentiation medium. Interestingly, the quantitative analysis of the ALP activity of OD-ECM-4 W with osteogenic differentiation medium increased more than 6 and 2 times compared to GM-ECM and OD-ECM-2 W (Fig. 3b). The results of the quantitatively measured ALP activity showed no significant differences among the groups at 4 weeks. The osteogenic differentiation medium of OD-ECM-2 W steadily increased during the 2 weeks.

### Calcification of re-seeded osteoblast on decellurized ECM matrix by alizarin red S staining and quantification

**Fig. 4:**
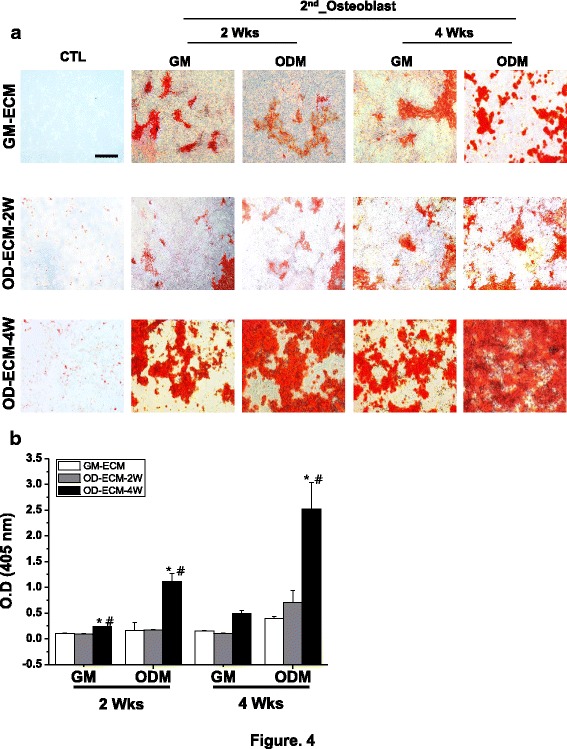
Alizarin red S staining and quantification on GM-ECM, OD-ECM-2 W, and OD-ECM-4 W for 2 and 4 weeks with growth media or osteogenic differentiation media. **a** Representative images of Alizarin red S staining with each decellularized ECMs and re-seeded osteoblast. The scale bar represents 400 μm. **b** Quantification of Alizarin red S with each group. ^*^p < 0.05 compared to GM-ECM, ^*#*^*p* < 0.05 compared to OD-ECM-2 W. CTL, control; GM, growth medium; ODM, osteogenic differentiation medium

The Alizarin red S staining is a stain commonly used stain to identify calcium containing osteocyte in differentiated osteoblast. The results of Alizarin S staining demonstrated that the control group did not indicate deposition of calcification by Alizarin S staining (Fig. 4a). To confirm the calcification of the re-seeded osteoblast, we stained the osteoblast on the various ECM matrices with Alizarin red S at different time points. After re-seeding the osteoblasts on GM-ECM and OD-ECM-2 W at 2 and 4 weeks, slightly increased calcium deposition in the growth medium and osteogenic differentiation medium was observed. The OD-ECM-4 W with growth medium and osteogenic differentiation medium showed more intense calcium deposition compared to GM-ECM and OD-ECM-2 W at 2 weeks. Additionally, the OD-ECM-4 W group with osteogenic differentiation medium showed vast extracellular calcium deposits and bright orange-red staining at 4 weeks. However, the measurement of the Alizarin red S staining showed a significant difference between the OD-ECM-4 W and other groups with growth medium and osteogenic differentiation medium at 2 weeks. The highest calcium deposition was observed in OD-ECM-4 W in osteogenic differentiation medium, which was 6 and 3 times higher than GM-ECM and OD-ECM-2 W at 4 weeks, respectively.

### Mineralization of re-seeded osteoblast on decellurized ECM matrix by von Kossa staining

**Fig. 5:**
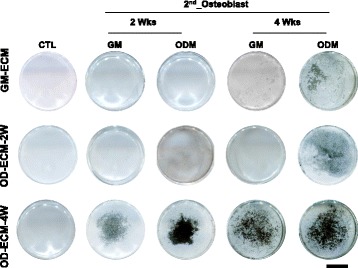
Representative images of von Kossa staining with various decellularized ECMs for 2 and 4 weeks with growth media or osteogenic differentiation media. The scale bar represents 10 mm

The von Kossa staining method is widely used to observe the presence of calcium phosphate. The von Kossa staining method involves a precipitated reaction in which silver ions react with phosphate, resulting in black precipitates. Mineralized cells can be seen easily with the naked eye after overall staining of the differentiated osteoblast of the culture layer. Deposition of calcium phosphate by von Kossa staining was not indicated in the GM-ECM, OD-ECM-2 W, and OD-ECM-4 W groups (Fig. 5). The intensity of von Kossa staining varied during the re-seeded osteoblast on various ECM matrixes. The re-seeded osteoblasts on OD-ECM-4 W with growth medium or osteogenic differentiation medium caused increased deposition of mineralization at 2 weeks. The strongest staining indication mineralization was observed OD-ECM-4 W with osteogenic differentiation medium at 4 weeks. Moreover, re-seeded osteoblasts on OD-ECM-4 W with growth medium confirmed highly mineralization compared to GM-ECM and OD-ECM-2 W at 4 weeks.

## Discussion

Decellularized ECM derived from in vitro cultured cells constructs offer an alternative to decellularized whole tissues for creating raw materials of tissue engineered scaffolds. Cell derived ECM is a naturally derived biomaterial formed by removed cellular components from original host cells. In this study, we prepared an effective decellularization process according to the selected methods, whereby the concentration of D-solution in the washing methods is optimized to preserve the ECM structure with the complete removal of cellular components. To optimize the complete decellularization of cells in vitro, a protocol for each specific step needs to be established cellular response, biochemical, and physiological characteristics. We attempted to optimize the complete decellularization of the cells using two different methods, freezing/thawing and osmotic pressure, using various solutions to complete decellularization (data not shown). The exact process used for decellularizing cultured cells is not well known yet because of the technical limitations of treatment time, cell density, treated D-solution. In this study, we used the osmotic pressure method to optimize the decellularization of cells using various treatment times, washing numbers, and concentrations of D-solution. This protocol was also used to prepare a completely decellularized ECM of osteogenic differentiated osteoblast cells, as evident on the immunohistochemistry and confirmed by DNA quantification.

For the evaluation of osteogenic differentiation, osteoblasts were re-seeded on various ECMs for analysis of commonly used staining and quantify the osteogenic differentiate related markers. ALP is one of the most commonly used early markers of osteogenesis and is also known to reflect the degree of osteogenic differentiation [24]. While ALP activity does not increase all groups in growth medium at 1 week, OD-ECM-4 W, which has a maturational osteogenic differentiated matrix, significantly increased in osteogenic differentiation medium at 1 week. The OD-ECM-4 W had the greatest ALP activity in the differentiation medium at 2 weeks compared to the other group with differentiation medium. These results indicate that the osteogenic differentiated osteoblast has a signal molecular remaining on the decellularized ECM that promoted early ALP activity. The ALP activity of GM-ECM and OD-ECM-2 W slightly increased at 4 weeks but showed no significant difference among these groups. This difference in osteogenic differentiation between the fully differentiated osteoblast as OD-ECM-4 W and the slightly differentiated osteoblast ECM as OD-ECM-2 W could be based on their different compositions as osteogenic transcription factor and organizations. In terms of osteoinductive properties, the mainly comprised of ECM which consisted mineralized component that remained comparatively unchanged after decellularization of fully osteogenic differentiated osteoblasts. Likewise, ALP activity generally coincides with the initiation of mineralization. We observed a rapid increase in ALP activity at 1 and 2 weeks of ODM and all OD-ECM-4 W in quantification of ALP activity.

Calcium deposition is a late stage osteogenic differentiation markers. We expect calcium content to increase over the culture period. Our results showed that OD-ECM-4 W with growth medium and osteogenic differentiation medium has significantly higher calcium deposition than the other group at 2 weeks. These results indicate that the fully differentiated osteoblasts ECM contains inorganic components that may have a synergistic effect with osteoblast differentiation. Many critical factors are still unknown for the optimal osteogenic differentiation using decellularized ECM. The Alizarin red S and von Kossa staining on OD-ECM-4 W with growth medium and osteogenic differentiation medium demonstrated that calcium deposition has a strong influence on osteogenic differentiation by mineralization. Clearly, the OD-ECM-4 W has a strong influence on osteogenic differentiation, but it is difficult to identify the specific components responsible. However, the influence of the OD-ECM-4 W with differentiation medium was well detected in all experiments via staining of ALP, Alizarin red S and von Kossa. We believe that osteogenic differentiation in vitro is affected by variations in osteogenic differentiation time and cell types. Consequently, the optimal differentiation protocol must be determined by preliminary experiments.

## Conclusions

We successfully decellularized osteoblast derived ECM with different composition and various differentiation times. The re-seeded osteoblasts on OD-ECM-4 W were promoted more osteogenic differentiation than that of other groups with growth medium. We revealed that OD-ECM is a promising osteogenic differentiated native platform for tissue engineering and stem cell culture applications.

## Abbreviations

ALP: alkaline phosphatase
CD-ECM: cell-derived ECM
CTL: control
DMEM: Dulbecco’s modified Eagles media
D-solution: decellularization solution
ECM: Extracellular matrix
FBS: fetal bovine serum
GM: growth medium
GM-EMC: normal osteoblast ECM
GM-Osteo: osteoblast cultured with growth medium
HA: hydroxyapatite
OD-ECM: osteogenic differentiated cell-derived ECM
ODM: osteogenic differentiation medium
OD-Osteo: osteoblast cultured with osteoblast differentiation medium
OPN: osteopontin
PBS: phosphate buffered saline
TCPS: tissue culture polystyrene
TD-ECM: tissue-derived ECM
TX: Triton X-100
